# Rice Yield and the Fate of Fertilizer Nitrogen as Affected by Addition of Earthworm Casts Collected from Oilseed Rape Fields: A Pot Experiment

**DOI:** 10.1371/journal.pone.0167152

**Published:** 2016-11-23

**Authors:** Min Huang, Xuefeng Zhou, Xiaobing Xie, Chunrong Zhao, Jiana Chen, Fangbo Cao, Yingbin Zou

**Affiliations:** Southern Regional Collaborative Innovation Center for Grain and Oil Crops (CICGO), Hunan Agricultural University, Changsha, China; University of Delhi, INDIA

## Abstract

The mechanism associated with improvement of soil nutritional status by oilseed rape crop, leading to better performance of rice crop, in rice-oilseed rape cropping systems is little known. The present study was aimed to test the hypothesis that earthworm casts produced during oilseed rape-growing season have positive effects on grain yield and fertilizer nitrogen (N) utilization in the subsequent flooded rice crop. A ^15^N-tracing pot experiment was conducted to determine the effects of earthworm casts collected from oilseed rape fields on yield attributes in rice and the fate of fertilizer N. Soil treated with earthworm casts (soil: earthworm casts = 4: 1, w/w) (EC_1_) produced 39% higher grain yield than soil only (EC_0_). EC_1_ had 18% more panicle number and 10% higher spikelet filling percentage than EC_0_. Aboveground biomass and harvest index were higher in EC_1_ than in EC_0_ by 20% and 15%, respectively. SPAD values in flag leaves were 10% and 22% higher under EC_1_ than EC_0_ at 15 and 20 days after heading, respectively. EC_1_ had 19% higher total N uptake and 18% higher physiological N-use efficiency than EC_0_. These positive effects of earthworm casts on yield attributes offset negative effects of decreasing N rate from 0.74 g pot^–1^ (equivalent to the recommended field rate of 150 kg ha^–1^) to 0.44 g pot^–1^ (equivalent to 60% of the recommended rate). Fertilizer N retention rate was 7% higher while fertilizer N loss rate was 6% lower in EC_1_ than in EC_0_. Our study suggests that earthworm casts produced during oilseed rape-growing season are expected to have the following benefits on the subsequent flooded rice system: (1) improving growth and physiological processes in rice plants and consequently increasing rice grain yield, and (2) increasing fertilizer N retention rate and hence decreasing fertilizer N loss rate and reducing environmental risk.

## Introduction

Rice is the staple food crop for a large segment of the world population [1]. China is one of the main rice production countries, and improving rice productivity in China is very important for world food security [2]. In the past five decades, rice yield has more than tripled in China [3]. Unfortunately, the increase in rice yield has been associated with a major decline in nutrient use efficiency, especially nitrogen (N) [4]. Agronomic N use efficiency in rice systems in China was 15–20 kg kg^–1^ in the early 1960s and declined to approximately 9 kg kg^–1^ in the early 1980s and to only about 6 kg kg^–1^ in the 2000s [5, 6, 7]. The low N use efficiency is mainly attributed to overfertilization [3, 4]. The average rate of N application for rice production in China is 180 kg ha^–1^, about 75% higher than the world average [3]. Because of the high rate of N application, only 20–30% of N is taken up by the rice plant and a large proportion of N is lost to the environment [7, 8]. The lost N has caused substantial environmental problems such as increased greenhouse gas emissions, enhanced N deposition and degradation of cropland and freshwater [4, 8, 9, 10].

Soil quality is critical to crop productivity and nutrient use efficiency [11]. Improving nutrient cycling is an important step towards stabilizing and optimizing soil quality [4]. Well-planned crop rotations, as compared to continuous monoculture systems, can be expected to promote nutrient cycling efficiency and consequently enhance crop productivity and reduce dependence on external fertilizer inputs [12, 13]. In China, rice-wheat and rice-oilseed rape are two long-established major rice-based rotation systems [14]. However, long-term experiments indicate that yields of rice-wheat cropping systems are stagnant or even declining [15]. What is worse, N fertilizer input has been excessively high for rice in the regions with rice-wheat rotations [8]. In Jiangsu, a typical rice-wheat cropping province in China, the average N rate for rice reaches 300 kg ha^–1^ in some counties [3]. By contrast, the N rate for rice is much lower in the regions with rice-oilseed rape cropping systems. In our long-term experiment, a high rice yield of around 10.0 t ha^–1^ was achieved at an N rate of 150 kg ha^–1^ in a rice-oilseed rape rotation [16, 17, 18]. This is related to the fact that oilseed rape is an excellent preceding crop–one that helps maintain or improve soil organic matter level, fauna abundance and aggregate stability.

Earthworms are arguably the most important components of the soil fauna in terms of soil fertility maintenance [19]. It is well documented that earthworms can improve soil nutrient cycling directly by consuming organic substrates and releasing nutrients into soils through metabolism processes, and indirectly by altering soil physical properties and creating distinctive structures (such as casts) [20]. In rice-oilseed rape rotations, although the earthworms usually migrate from the field during rice-growing season due to water flooding, the earthworm casts produced during oilseed rape-growing season remain in the field. Here, we hypothesized that the earthworm casts present in the agricultural soil may have positive effects on grain yield and fertilizer N utilization in the subsequent flooded rice crop. To test this hypothesis, a ^15^N-tracing pot experiment was conducted to determine the effects of earthworm casts collected from oilseed rape fields on yield attributes in rice and the fate of fertilizer N.

## Materials and Methods

### Ethics statements

No specific permissions were required for the activities conducted in this study. The fields are neither privately owned nor protected. The experiments did not involve endangered or protected species.

### Experimental details

**Fig 1:**
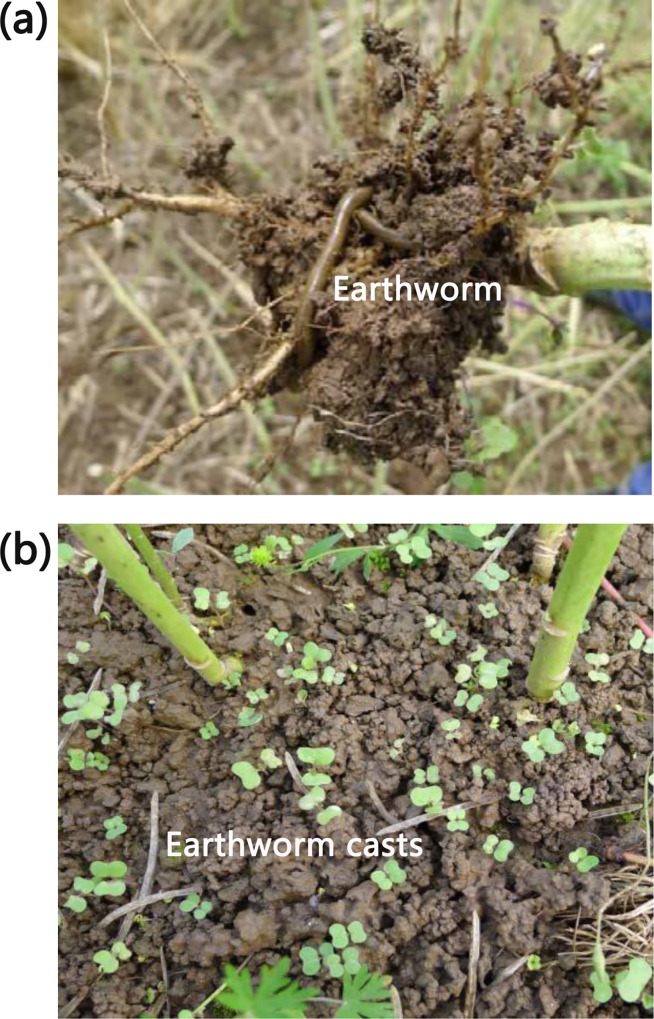
Oilseed rape fields for collecting earthworm casts in this study. (a) An earthworm in root-adhering soil of oilseed rape and (b) a pile of earthworm casts in an oilseed rape field.

An outdoor pot experiment was conducted at the research farm of Hunan Agricultural University (28°11′N, 113°04′E), Changsha, Hunan Province, China in single rice-growing season (from May to October) in 2015. The soil used in the experiment, classified as an Ultisol (USDA taxonomy), was collected from the upper 20 cm of a rice paddy at the research farm. The tested earthworm casts was collected from oilseed rape fields located at Nanxian (29°21′N 112°25′E), Hunan Province (Fig 1). The chemical properties of the soil and earthworm casts were analyzed and shown in Table 1. The pH was determined by a digital pH meter, organic matter by the potassium dichromate method, total N by the semi-micro Kjeldahl method, available P by the Olsen method, and available K by an atomic absorption spectrophotometry [21]. The soil and earthworm casts were air-dried and sieved (5 mm) before use.

**Table 1:** Chemical properties of soil and earthworm casts used in this study.

Property	Soil	Earthworm casts
pH	5.8	8.2
Organic matter (g kg^–1^)	27.6	79.5
Total N (g kg^–1^)	1.6	2.4
Available P (mg kg^–1^)	54.5	3.0
Available K (mg kg^–1^)	63.2	240.6

Liangyoupeijiu, an elite hybrid rice cultivar, was grown under a factorial combination of two levels of earthworm casts and two N rates, with each combination replicated five times. The two levels of earthworm casts were 0% (soil only) and 20% (soil: earthworm casts = 4: 1, w/w), which were denoted as EC_0_ and EC_1_, respectively. The soil or the mixture of soil and earthworm casts was filled in plastic pots (diameter = 23 cm, height = 25 cm) with an amount of 7 kg pot^–1^. The two N rates were 0.74 (N_1_) and 0.44 g N pot^–1^ (N_2_), equivalent to the recommended field rate of 150 kg N ha^–1^ and 60% of the recommended rate (90 kg N ha^–1^), respectively.

Rice seeds were treated with a seed coating with fungicide and soaked in sterilized water for 24 h at room temperature. The soaked seeds were kept between thick layers of cotton cloth and allowed to germinate at 38°C. Pre-germinated seeds were sown in seedling trays. Twenty five-day-old seedlings were transplanted into the pots with one seedling per pot. Fertilizers used were ^15^N-labeled urea (5.18% isotopic abundance, provided by Shanghai Institute of Chemical Industry, China) for N, single superphosphate for P (0.29 g P_2_O_5_ pot^–1^, equivalent to 60 kg P_2_O_5_ ha^–1^) and potassium chloride for K (0.52 g K_2_O pot^–1^, equivalent to 105 kg K_2_O ha^–1^). N was split-applied: 50% at basal (1 day before transplanting), 30% at early tillering (7 days after transplanting), and 20% at panicle initiation. P was applied at basal. K was split equally as basal and top dressing at the panicle initiation. A floodwater depth of approximately 5 cm was maintained during the whole growth period. Insects and diseases were controlled by chemicals, and weeds were removed by hand.

Three pots with uniform plants were selected for each treatment. SPAD value (SPAD 502 portable chlorophyll meter, Minolta Camera Co., Osaka, Japan) in flag leaf of main stem was determined from 5 to 20 days after heading at a 5-day interval according to the procedures in Peng et al. [22]. At maturity, a mixed soil sample was taken from 5 randomly selected points within each pot, throughout the whole soil profile, using a 1.8-cm inner diameter tube auger. Aboveground parts were sampled and separated into straw and panicles. Panicles were counted and hand-threshed. Filled spikelets were separated from unfilled spikelets by submerging them in tap water, and number of filled and unfilled spikelets was counted. Dry weights of straw, rachis, and filled and unfilled spikelets were determined after oven drying at 70°C to constant weight. Spikelets per panicle, spikelet filling percentage, grain weight, aboveground biomass and harvest index were calculated. Total N concentrations and ^15^N abundances in soil and plant samples were determined with a VAP50 Kjeldahl meter (Gerhardt, Königswinter, Germany) and a Delta V Advantage isotope mass spectrometer (Thermo Fisher, Waltham, MA, USA), respectively. Total N uptake, N uptake from fertilizer and physiological N-use efficiency in rice as well as fertilizer N retention, recovery and loss rates were calculated according to Huang et al. [23]. Grain yield was adjusted to a standard moisture content of 14%.

### Statistical analysis

All data were analyzed by analysis of variance (Statistix 8.0, Analytical software, Tallahassee, FL, USA). The statistical model included replication, earthworm cast treatment, N rate, and the interaction between earthworm cast treatment and N rate.

## Results

**Fig 2:**
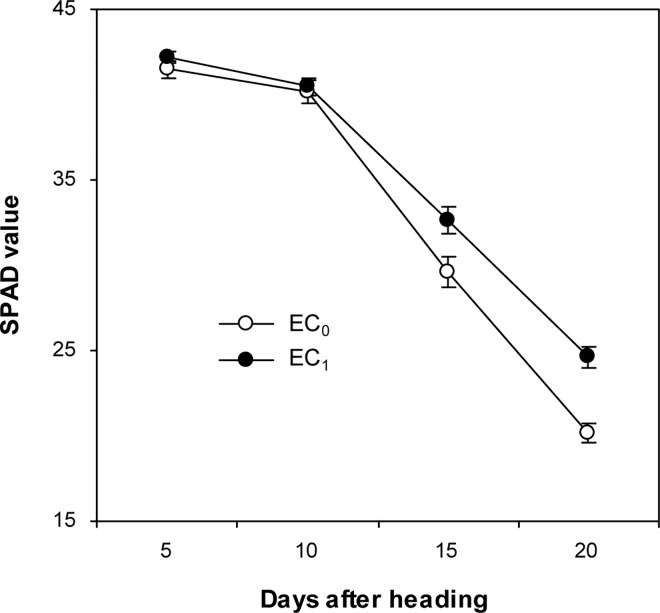
Effects of addition of earthworm casts on SPAD values in flag leaves in rice. EC_0_, soil only; EC_1_, soil: earthworm casts = 4: 1 (w/w). Data are the means across two N rates. Vertical bars represent SE (*n* = 6).

EC_1_ produced 39% higher grain yield than EC_0_ (Table 2). EC_1_ had 18% more panicle number and 10% higher spikelet filling percentage than EC_0_. There were no significant differences in spikelet number per panicle and grain weight between EC_1_ and EC_0_. Aboveground biomass and harvest index were 20% and 15% higher in EC_1_ than in EC_0_, respectively. Grain yield was 17% lower under N_2_ than under N_1_. N_2_ had 13% less panicle number but 5% higher grain weight than N_1_. The differences in spikelet number per panicle and spikelet filling percentage were not significant between N_2_ and N_1_. Aboveground biomass and harvest index were 10% and 9% lower under N_2_ than N_1_, respectively. The interactive effects of earthworm cast and N rate treatments on all the yield attributes were not significant. Also because of this, mean data across two N rates were presented for SPAD values in flag leaves (Fig 2). There were no significant differences in SPAD values in flag leaves between EC_0_ and EC_1_ at 5 and 10 days after heading, while at 15 and 20 days after heading SPAD values in flag leaves were 10% and 22% higher under EC_1_ than EC_0_, respectively.

**Table 2:** Effects of addition of earthworm casts on yield attributes in rice under two N rates.

Treatment		Grain yield (g pot^–1^)	Panicles pot^–1^	Spikelets panicle^–1^	Spikelet filling (%)	Grain weight (mg)	Aboveground biomass (g pot^–1^)	Harvest index
Earthworm cast^a^	N rate^b^							
EC_0_	N_1_	64.7 (2.5)^c^	18.7 (0.7)	174 (5)	75.5 (0.9)	26.4 (0.7)	134 (4)	0.42 (0.01)
	N_2_	55.2 (2.5)	16.3 (0.7)	168 (7)	72.0 (2.7)	28.0 (0.6)	120 (2)	0.40 (0.02)
	Mean	59.9	17.5	171	73.8	27.2	127	0.41
EC_1_	N_1_	92.4 (0.2)	22.0 (1.2)	185 (6)	86.9 (1.0)	26.2 (0.4)	159 (2)	0.50 (0.01)
	N_2_	74.6 (5.6)	19.3 (1.2)	173 (4)	81.5 (4.1)	27.4 (0.4)	145 (6)	0.44 (0.02)
	Mean	83.5	20.7	179	84.2	26.8	152	0.47
Analysis of variance								
Earthworm cast		**	**	ns	**	ns	**	**
N rate		**	**	ns	ns	*	**	*
Earthworm cast × N rate		ns	ns	ns	ns	ns	ns	ns

^a^EC_0_, soil only; EC_1_, soil: earthworm casts = 4: 1 (w/w).

^b^N_1_, 0.74 g N pot^–1^ (equivalent to 150 kg N ha^–1^); N_2_, 0.44 g N pot^–1^ (equivalent to 90 kg N ha^–1^).

^c^Values in parentheses are SE (*n* = 3).

**, significance at 0.01 probability level

*, significance at 0.05 probability level; ns, non-significance at 0.05 probability level.

The difference in N uptake from fertilizer between EC_1_ and EC_0_ was insignificant (Table 3). EC_1_ had 19% higher total N uptake and 18% higher physiological N-use efficiency than EC_0_. N uptake from fertilizer and total N uptake N rate were lower under N_2_ than under N_1_ by 42% and 10%, respectively. The difference in physiological N-use efficiency was not significant between N_2_ and N_1_. There were no significant interactive effects of earthworm cast and N rate treatments on N uptake from fertilizer, total N uptake and physiological N-use efficiency.

**Table 3:** Effects of addition of earthworm casts on N uptake and physiological N-use efficiency in rice under two N rates.

Treatment		N uptake (g pot^–1^)		Physiological N-use efficiency (g g^–1^)
Earthworm cast^a^	N rate^b^	From fertilizer	Total	
EC_0_	N_1_	0.18 (0.02)^c^	0.89 (0.03)	72.5 (1.5)
	N_2_	0.11 (0.01)	0.80 (0.02)	69.1 (2.8)
	Mean	0.14	0.84	70.8
EC_1_	N_1_	0.18 (0.01)	1.05 (0.03)	88.3 (2.6)
	N_2_	0.10 (0.01)	0.95 (0.05)	79.0 (5.7)
	Mean	0.14	1.00	83.6
Analysis of variance				
Earthworm cast		ns	**	*
N rate		**	*	ns
Earthworm cast × N rate		ns	ns	ns

^a^EC_0_, soil only; EC_1_, soil: earthworm casts = 4: 1 (w/w).

^b^N_1_, 0.74 g N pot^–1^ (equivalent to 150 kg N ha^–1^); N_2_, 0.44 g N pot^–1^ (equivalent to 90 kg N ha^–1^).

^c^Values in parentheses are SE (*n* = 3).

**, significance at 0.01 probability level

*, significance at 0.05 probability level; ns, non-significance at 0.05 probability level.

EC_1_ had 7% higher fertilizer N retention rate but 6% lower fertilizer N loss rate than EC_0_ (Table 4). There was no significant difference in fertilizer N recovery rate between EC_1_ and EC_0_. N_2_ had 6% higher fertilizer N retention rate but 4% lower fertilizer N loss rate than N_1_. There was no significant difference in fertilizer N recovery rate between N_2_ and N_1_. The interactive effects of earthworm cast and N rate treatments on fertilizer N retention, recovery and loss rates were insignificant.

**Table 4:** Effects of addition of earthworm casts on fate of fertilizer-N applied to rice under two N rates.

Treatment		Retention rate (%)	Recovery rate (%)	Loss rate (%)
Earthworm cast^a^	N rate^b^			
EC_0_	N_1_	14.9 (0.1)^c^	29.5 (1.7)	55.6 (1.8)
	N_2_	20.4 (0.9)	28.1 (0.9)	51.4 (1.4)
	Mean	17.7	28.8	53.5
EC_1_	N_1_	21.5 (1.4)	29.0 (1.3)	49.5 (1.4)
	N_2_	28.6 (2.8)	26.5 (0.7)	44.8 (2.0)
	Mean	25.1	27.8	47.2
Analysis of variance				
Earthworm cast		**	ns	**
N rate		**	ns	*
Earthworm cast × N rate		ns	ns	ns

^a^EC_0_, soil only; EC_1_, soil: earthworm casts = 4: 1 (w/w).

^b^N_1_, 0.74 g N pot^–1^ (equivalent to 150 kg N ha^–1^); N_2_, 0.44 g N pot^–1^ (equivalent to 90 kg N ha^–1^).

^c^Values in parentheses are SE (*n* = 3).

**, significance at 0.01 probability level

*, significance at 0.05 probability level; ns, non-significance at 0.05 probability level.

## Discussion

Oilseed rape has a useful soil improving role that aided the performance of subsequent crops. However, this role is not fully understood in rice-oilseed rape cropping systems. In present study, we determined the responses of rice to addition of earthworm casts collected from oilseed rape fields. Our results showed that grain yield increased by addition of earthworm casts. In this regard, it was reported that earthworm casts have a significant positive influence on rice yield through the creation of patches of fertility [24]. Consistently, in the present study, soil organic matter, total N and available K concentrations were 2.88, 1.50 and 3.81 times higher in the earthworm casts than in the soil, respectively (Table 1). Furthermore, our results showed that addition of earthworm casts increased total N uptake but did not affect N uptake from fertilizer by rice. These demonstrate that the positive effect of addition of earthworm casts on grain yield was driven by its high fertility but not by its impact on fertilizer N utilization.

Prior to this study, there was limited information available on the growth and physiological processes regarding the positive effect of earthworm casts on rice yield. Our results showed that the increased panicle number and spikelet filling percentage were achieved without sacrificing spikelet number per panicle and grain weight under addition of earthworm casts. In rice crops, compensations between yield components are always arising from either physiological competition or developmental allometry [16]. Typically, there is usually a negative relationship between panicle number and spikelet number per panicle [16, 25]. However, in this study, the increased panicle number did not result in the decline of spikelet number per panicle under addition of earthworm casts. In this regard, it is reported that increasing biomass production plays an important role in detaching the compensation between panicle number and spikelet number per panicle in rice [26]. Therefore, in this study, the increased aboveground biomass was responsible for the increased panicle number without the decline of spikelet number per panicle under addition of earthworm casts. Also, partly because of the increased aboveground biomass, the more number of spikelets was achieved not at the expense of spikelet filling percentage and grain weight under addition of earthworm casts. Although it is generally accepted that improvement in rice yield may be driven from the increased biomass production rather than harvest index [16, 25, 26], there have been reports showing that raising rice yield is possible by increasing harvest index [27, 28]. In the present study, the increased rice yield under addition of earthworm casts was attributed to increases in both biomass production and harvest index. Biomass production can be increased by increasing growth duration or crop growth rate or both [29]. Because growth duration was nearly not affected by addition of earthworm casts, the increased biomass production under addition of earthworm casts was attributed to increased crop growth rate. Crop growth rate is a function of canopy gross photosynthesis and crop respiration [30]. SPAD values in flag leaves were increased by addition of earthworm casts at 15 and 20 days after heading, indicating that a greater single-leaf photosynthetic rate during the middle and late grain filling period might be partly responsible for the higher crop growth rate under addition of earthworm casts. Harvest index is determined by the potential sink size, by the transient photosynthesis during grain formation, and by the remobilization of stored reserves into the growing grain [31]. In the present study, the more number of spikelets and the greater single-leaf photosynthetic rate during the middle and late grain filling period might be partially responsible for the increased harvest index under addition of earthworm casts. Moreover, it has been reported that increase in harvest index is beneficial to increase the physiological N-use efficiency in rice [28]. Consistently, in this study, the physiological N-use efficiency was increased by addition of earthworm casts.

Although addition of earthworm casts did not affect fertilizer N recovery rate, it increased fertilizer N retention rate. In other words, soil fertility could be indirectly improved by addition of earthworm casts through increasing fertilizer N retention. This result is in agreement with that reported by Groffman et al. [32], who observed that earthworm activity increased soil microbial biomass carrying capacity and N retention. As a consequence of the increased fertilizer N retention rate, fertilizer N loss rate declined due to addition of earthworm casts, suggesting that earthworm casts have beneficial effects in reducing environmental risk. It is well known that fertilizer N applied to rice paddies can be lost through denitrification, ammonia (NH_3_) volatilization, surface runoff, and leaching [7]. In this study, N lost by surface runoff and leaching can be excluded, since the water level was controlled to avoid overtopping and the pots used were closed at the bottom. Therefore, the N loss in the present study was presumed to be due to denitrification and NH_3_ volatilization. There has been report showing that nitrous oxide production is higher in earthworm casts than soils under anaerobic conditions [33]. Accordingly, it seemed that N loss through denitrification might not be reduced by addition of earthworm casts in this study, but further investigations are needed to confirm this speculation. Moreover, this study does not allow us to draw a concrete conclusion on the effect of addition of earthworm casts on NH_3_ volatilization. On one hand, earthworm casts had higher soil pH (Table 1). Soil pH was increased from 5.8 to 7.0 by addition of earthworm casts (data not shown). In general, NH_3_ volatilization is increased with increasing soil pH [34]. Considered from this point of view, addition of earthworm casts has potential to increase NH_3_ volatilization. But on the other hand, earthworm casts had higher C:N ratio (Table 1), which prevents N loss through NH_3_ volatilization [35]. These highlight the need for greater fundamental understanding of the effects of earthworm casts on pathways of fertilizer N loss in rice paddies.

Reducing fertilizer N inputs has become increasingly attractive in rice production in China because only by it can degraded environments be gradually restored, enhanced and protected [8]. Our study showed that decreasing N rates from 0.74 g pot^–1^ (equivalent to the recommended field rate of 150 kg ha^–1^) to 0.44 g pot^–1^ (equivalent to 60% of the recommended rate) resulted in lower fertilizer N loss rate. However, the decrease in N rate also caused reductions in N uptake, panicle number, aboveground biomass and harvest index, and consequently lower grain yield. Huang et al. [36] determined the N response of two rice cultivars, including the one (Liangyoupeijiu) used in this study, over a wide range of N rates (60–410 kg ha^–1^). They found that both cultivars required a minimum total N rate of 120–150 kg ha^–1^ to produce maximum grain yield. These indicate that amount of reducing fertilizer N should be properly selected in order to avoid yield loss. But more interestingly, our results showed that the negative effect of decreasing N rate on grain yield could be offset by the positive effect of addition of earthworm casts (Table 2). This suggests that any agricultural system promoting earthworm development, thereby increasing the earthworm casts, could be a useful approach in reducing fertilizer N inputs in rice production. Rice-oilseed rape rotation appears to be such an agricultural system. In our on-farm investigations in 2015 and 2016, earthworm densities were 1.65–2.54 times higher in fields with rice-oilseed rape than with rice-fallow cropping systems during the upland period (data not shown). This might be partly responsible for the relatively lower N rate for rice production in the regions with rice-oilseed rape rotations.

Taken together, it is expected that earthworm casts produced during oilseed rape-growing season have the following benefits on the subsequent flooded rice system: (1) improving growth and physiological processes in rice plants and consequently increasing rice grain yield, and (2) increasing fertilizer N retention rate and hence decreasing fertilizer N loss rate and reducing environmental risk. However, there are some limitations in the study that must be acknowledged. Firstly, because the study was conducted under pot conditions, the results are not necessarily applicable to field conditions. Secondly, the addition rate of earthworm casts in the study is not exactly equal to the amount of earthworm casts in the fields. Therefore, further studies are needed to replicate the study under field conditions.
